# Circ_UBE2D2 Attenuates the Progression of Septic Acute Kidney Injury in Rats by Targeting miR-370-3p/NR4A3 Axis

**DOI:** 10.4014/jmb.2112.12038

**Published:** 2022-05-11

**Authors:** Yanghui Huang, Guangyu Zheng

**Affiliations:** 1 Emergency Medicine Department, Clinical Medical College and Affiliated Hospital of Chengdu University, Sichuan Province, 610081, P.R. China; 2 Emergency Medicine Department, The First People’s Hospital of Yibin, Yibin City, Sichuan Province 644000, P.R. China

**Keywords:** Circ_UBE2D2, MiR-370-3p, NR4A3, septic acute kidney injury, apoptosis

## Abstract

As circ_UBE2D2 has been confirmed to have targeted binding sites with multiple miRNAs involved in septic acute kidney injury (SAKI), efforts in this study are directed to unveiling the specific role and relevant mechanism of circ_UBE2D2 in SAKI. HK-2 cells were treated with lipopolysaccharide (LPS) to construct SAKI model in vitro. After sh-circ_UBE2D2 was transfected into cells, the transfection efficiency was detected by qRT-PCR, cell viability and apoptosis were determined by MTT assay and flow cytometry, and expressions of Bcl-2, Bax and Cleaved-caspase 3 were quantified by western blot. Target genes associated with circ_UBE2D2 were predicted using bioinformatics analysis. After the establishment of SAKI rat model, HE staining and TUNEL staining were exploited to observe the effect of circ_UBE2D2 on tissue damage and cell apoptosis. The expression of circ_UBE2D2 was overtly elevated in LPS-induced HK-2 cells. Sh-circ_UBE2D2 can offset the inhibition of cell viability and the promotion of cell apoptosis induced by LPS. Circ_UBE2D2 and miR-370-3p as well as miR-370-3p and NR4A3 have targeted binding sites. MiR-370-3p inhibitor reversed the promoting effect of circ_UB2D2 silencing on viability of LPS-treated cells, but shNR4A3 neutralized the above inhibitory effect of miR-370-3p inhibitor. MiR-370-3p inhibitor weakened the down-regulation of NR4A3, Bax and Cleaved caspase-3 and the up-regulation of Bcl-2 induced by circ_UB2D2 silencing, but these trends were reversed by shNR4A3. In addition, sh-circ_UBE2D2 could alleviate the damage of rat kidney tissue. Circ_UBE2D2 mitigates the progression of SAKI in rats by targeting miR-370-3p/NR4A3 axis.

## Introduction

Sepsis is a disease characterized by systemic inflammatory response syndrome (SIRS) resulted from bacterial, viral or fungal infections [1, 2]. The host response to infection causes multiple organ failure, with the kidney being one of the most commonly affected organs [3, 4]. One of the most serious and common complications of sepsis is sepsis acute kidney injury (SAKI) [5, 6]. Sepsis is associated with up to 50% of acute kidney injury (AKI), and over 60% of patients with sepsis have AKI [6] whose mortality rate exceeds that of sepsis patients without AKI [7]. Recent studies have verified that the pathogenesis of sepsis-induced AKI includes a series of complex interactions between vascular endothelial cell dysfunction, inflammation and renal tubular cell apoptosis [8, 9]. However, efforts to translate these findings from the laboratory to the clinic in clinical trials have proven to be a failure. Therefore, it is necessary to comprehensively interpret the pathogenesis of septic renal injury in order to develop more effective treatment strategies.

Circular ribonucleic acid (circRNA) is a kind of widely existing non-coding ribonucleic acid, which has a closed-loop structure and high stability [10, 11]. There is increasing evidence showing that many circRNAs are closely related to a variety of human diseases, including sepsis [12, 13]. However, limited attention has been paid to the role of circRNA in SAKI, which needs to be supplemented and improved. According to the relevant studies about the impacts of circRNA on sepsis-associated diseases, circ_0114428 was confirmed to regulate sepsis-induced kidney injury by targeting the miR-495-3p/CRBN axis [14], and circular RNA TLK1 promotes sepsis-associated AKI by modulating inflammation and oxidative stress through miR-106a-5p/HMGB1 axis [15]. CircRNA ubiquitin-conjugating enzyme E2 D2 (circ_UBE2D2) is a newly identified RNA molecule [16]. In retrospect of existing literature, we found that miR-942-5p, miR-122-3p, miR-370-3p and miR-337 are implicated in SAKI process [17-20], and circ_UBE2D2 has targeted binding sites with these miRNAs . However, the role of circ_UBE2D2 in SAKI has not been discussed. Whether it can affect the SAKI process through endogenous competitive binding with miRNAs is worth further exploring.

NR4A3, a member of nuclear receptor subfamily 4, is an important regulator of cellular function and inflammation [21]. Studies have evidenced that NR4A3 is a pro-apoptotic gene which is strongly induced and expressed in AKI [22, 23]. Through predicting target miRNAs of circ_UBE2D2 and NR4A3 by StarBase and miRDB respectively, we uncovered that they can bind to miR-370-3p together. Thus, we speculated that circ_UBE2D2 may affect the process of septic kidney injury by targeting miR-370-3p to promote the expression of NR4A3.

## Methods

### Ethical Statement

The animal experiment in this study was approved by the Animal Ethics Committee of Nanfang Hospital.

### Cell Culture

Renal tubular epithelial cells HK-2 were purchased from the Cell bank of Chinese Academy of Sciences (China). All cells were cultured in DMEM/F12 medium containing 10% fetal bovine serum and cultured in a humidified atmosphere at 37°C with 5% CO_2_. To establish a SAKI model in vitro, HK-2 cells were treated with 10 μg/ml lipopolysaccharide (LPS, L2630, Sigma, USA) [24, 25].

### Cell Transfection

HK-2 cells were inoculated into a 24-well plate and cultured at 37°C in a 5% CO_2_ incubator. MiR-370-3p inhibitor (miR20000722-1-5) and miR-370-3p inhibitor negative control (NC, miR2N0000001-1-5) were obtained from RiboBio (China). When the cell confluence reached 80% under an inverted microscope, miR-370-3p inhibitor, negative control, as well as short hairpin RNA against circ_UBE2D2 (shRNA-circ_UBE2D2) and shNR4A3 lentiviruses were transfected into cells that were cultured with DMEM containing 10% fetal bovine serum in a 24-well plate. After the culture medium was refreshed, the culture was continued for 3 days and cells were collected for subsequent experiments.

### Dual-Luciferase Reporter Assay

Targeted binding sites of circ_UBE2D2 or NR4A3 to miR-370-3p were predicted by StarBase (http://starbase.sysu.edu.cn/) and miRDB. The 3'UTR sequences of circ_UBE2D2 or NR4A3 were obtained from NCBI (https://www.ncbi.nlm.nih.gov/). Then, the 3'UTR of wild-type or mutant circ_UBE2D2 or NR4A3 (circ_UBE2D2-WT/NR4A3-WT, circ_UBE2D2-MUT/NR4A3-MUT) was separately cloned into pmirGLO vector (E1330, Promega, China). HK-2 cells were co-transfected with circ_UBE2D2-WT/NR4A3-WT/circ_UBE2D2-MUT/NR4A3-MUT and miR-370-3p mimic/mimic control under the help of Lipo3000. The transfection process was referred to cell transfection above. Dual-luciferase reporter detection system (E1910, Promega, China) was used to detect luciferase activity of each group 48 h after transfection.

### 3-(4,5)-Dimethylthiahiazo (-z-y1)-3,5-di- Phenytetrazoliumromide (MTT) Assay

Cell suspension containing about 2 × 10^3^ HK-2 cells was transferred to the 96-well plate at 100 μl/well. 10 μl MTT reagent (ChemicalBook, China) was added to each well, and incubated at 37°C for 1 h. The absorbance value at 450 nm was determined by a microplate reader (BIO-RAD550, Bio-Rad, USA).

### Flow Cytometry

2× 10^3^ HK-2 cells were seeded into each well of the 6-well plate, and then the cells were digested by trypsin to prepare single-cell suspension for cell apoptosis detection. The single-cell suspension was reacted with Annexin V-FITC staining solution and propidium iodide (PI) staining solution for 5 minutes (min) in the dark. Ultimately, a flow cytometer was exploited to detect cell apoptosis.

### Animal Source and Grouping

Twenty Wistar rats (weight: 200-220 g) purchased from SLAC Laboratory Animal Co., Ltd, were reared in cages under standard temperature and humidity with a 12 h light/dark cycle. Animal experiments were carried out in accordance with the Regulations of the Peoplés Republic of China on the Administration of Experimental Animals and the Guiding Opinions on the Ethical Treatment of Experimental Animals. The rats were randomly divided into 4 groups with 5 rats in each group, which were named as control group (rats were treated with sham surgery only), cecal ligation and puncture (CLP) group (rats were subjected to AKI surgery), CLP+shNC group (rats were treated with AKI surgery and injected with shNC) and CLP+ sh-cirC_UBE2D2 group (rats underwent AKI surgery and were injected with sh-cirC_UBE2D2).

### Establishment of SAKI Rat Model

For the establishment of SAKI rat model, the operation involved exposing the cecum, followed by ligation and perforation. Thereafter, the rats were placed in a warm environment to recover. Before sample collection (rats in the control group were intraperitoneally injected with pentobarbital 60 mg/kg immediately after intramuscular injection of normal saline), rats in each group were deeply anesthetized. The abdominal cavity was opened through a median incision, and blood samples were taken from the inferior vena cava, and then centrifuged at 3,000 r/min for 10 min to separate the serum. The levels of blood urea nitrogen (BUN) and serum creatinine (Scr) in serum were measured using a MedLab automated biomedical analyzer (Nanjing medease science and technology corporation, China). The rats were sacrificed by cervical dislocation. Both kidneys were extracted quickly, and the right kidney was stored at -80°C for western blot experiment. The renal tissue was fixed with 4%paraformaldehyde for subsequent hematoxylin-eosin (HE) staining.

### HE Staining

The tissue fixed by fixative solution was cut into sections with a thickness of 5 µm. After routine dewaxation and hydration, the tissue sections were stained with hematoxylin and eosin, and finally sealed with neutral gum. The tissue sections were imaged under a Motic-6.0 Image acquisition system, and tissue damage was analyzed.

### TUNEL Staining

Other tissue sections were used for TUNEL staining. The number of cells in the kidney tissue and that of positive cells were counted under the microscope. The ratio of positive cell number to cell number in the kidney tissue was calculated, which was defined as the cell apoptosis rate.

### Quantitative Reverse Transcription Polymerase Chain Reaction (qRT-PCR)

Total RNA was extracted from cells and tissues by Trizol reagent (12183555, Thermo Fisher Scientific, USA) and then reverse-transcribed into complementary (c)DNA (cDNA synthesis kit, Takara) according to the instructions of kit. Power SYBR Green PCR Master Mix (Takara) was used for real-time PCR with an ABI7500 System (Applied Biosystems) as per the manufacturer's specification. The gene expression was quantified using 2^-ΔΔCT^ method [26]. The RNA primer sequences are listed in Table 1. β-actin was used as an internal reference.

### Western Blot

Cells and tissues were collected and lysed with Cell Lysis Buffer (R0278, Sigma-Aldrich, USA) to obtain total protein. The protein content of the sample was determined by BCA kit (55R-1544, Fitzgerald, USA) at 562 nm. Then, total protein was isolated with 12% sodium dodecyl sulfate-polyacrylamide gel electrophoresis (SDS-PAGE), transferred to PVDF membrane (24937, Sigma-Aldrich, China), and sealed with 5% skim milk powder at room temperature for 2 h. The sealed membrane was washed with Tris Buffered Saline with Tween (TBST) for 3 times. Subsequently, the membrane was reacted with the primary antibodies at 4°C overnight. On the next day, the membrane was washed with TBST for 3 times, followed by the incubation with secondary antibodies at room temperature for 2 h. The protein bands were detected by chemiluminescence (Sinopharm Chemical Reagent Co. Ltd, China) and photographed by gel imaging system. The gray value of the target protein is divided by the gray value of the internal reference β-actin to correct the error, and the obtained result represents the relative content of the target protein in a sample. The primary antibodies used are those against NR4A3 (ab259939, Abcam), Bcl-2 (ab32124, Abcam), Bax (ab32503, Abcam), Cleaved caspase-3 (ab32042, Abcam) and β-actin (ab8226, Abcam).

### Statistical Analysis

**Fig. 1:**
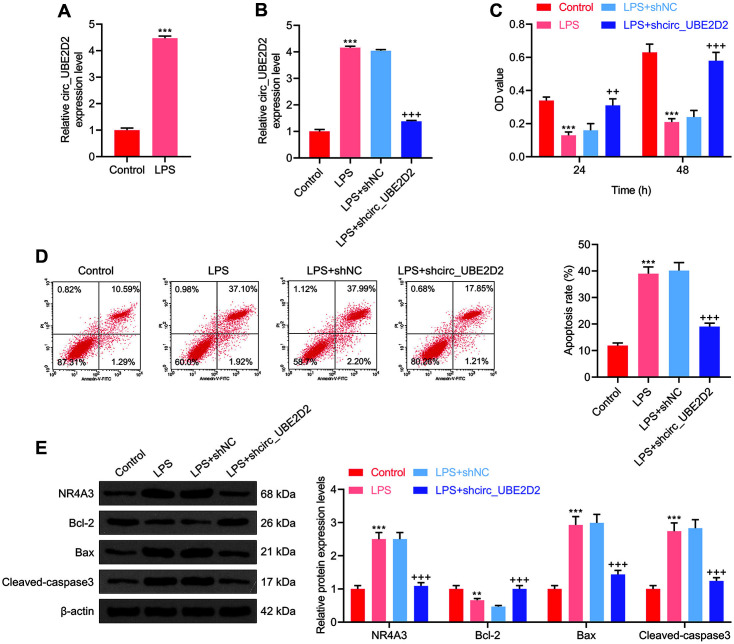
Effects of circ_UBE2D2 on LPS-induced renal tubular epithelial cell viability and apoptosis. (**A**) QRTPCR was used to detect the expression of circ_UBE2D2 in LPS-induced HK-2 cells. (**B**) The expression of circ_UBE2D2 in cells transfected with sh-circ_UBE2D2 vector was detected by qRT-PCR. (**C**) MTT assay was used to assess the effect of circ_UBE2D2 on LPS-induced cell viability. (**D**) Flow cytometry was applied to evaluate the effect of circ_UBE2D2 on LPSinduced apoptosis of HK-2 cells. (**E**) Western blot was exploited to determine the effect of circ_UBE2D2 on LPS-induced HK- 2 cell apoptosis-related protein and NR4A3 expressions. (**p < 0.01, ***p < 0.001, vs. Control; ^++^*p* < 0.01, ^+++^*p* < 0.001, vs. LPS+shNC).

**Fig. 2:**
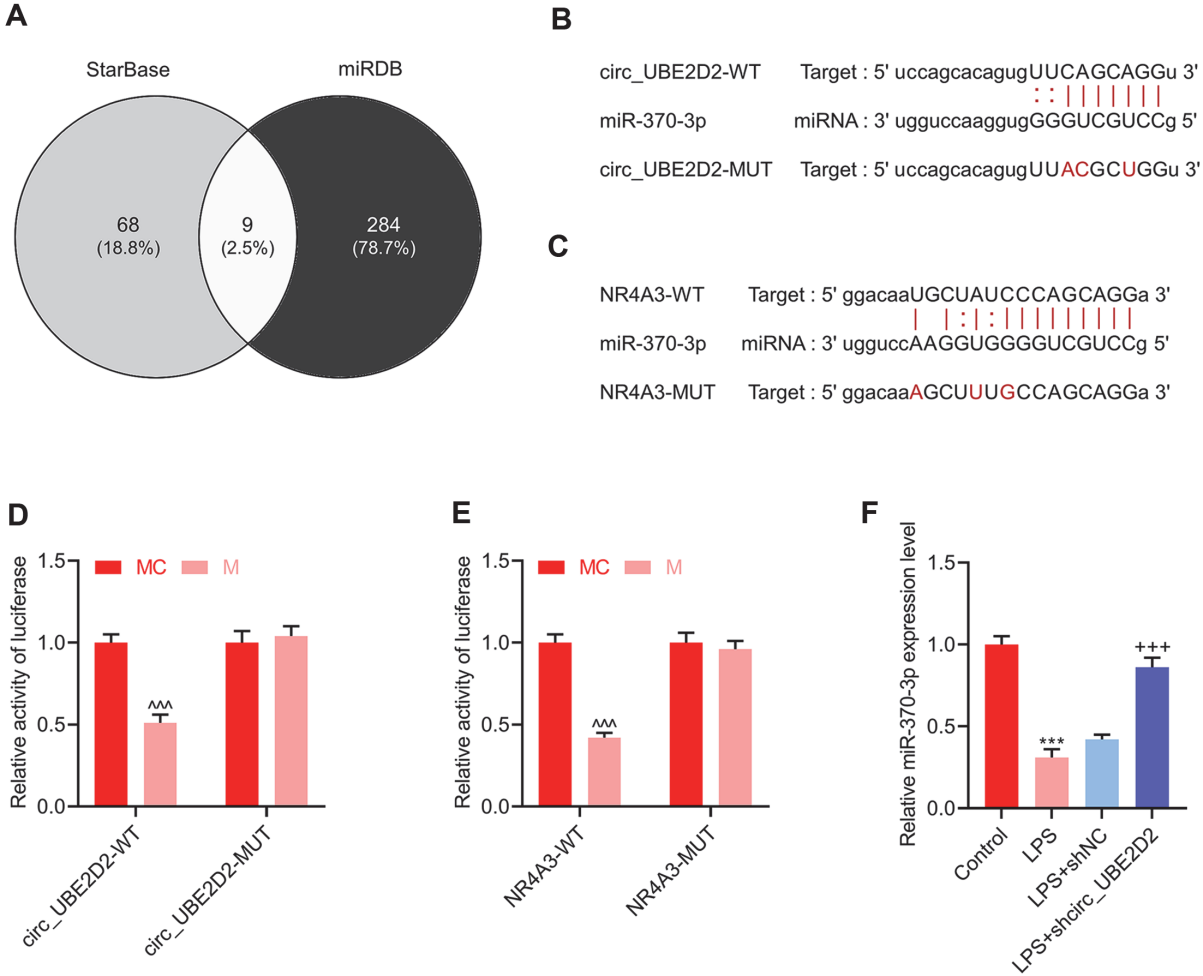
Prediction analysis of circ_UBE2D2 target gene. (**A**) StarBase and miRDB predict the binding miRNAs of circ_UBE2D2 and NR4A3, respectively, and the co-targeting miRNA with the highest score (miR-370-3p) was selected as the research object. (**B**) StarBase was used to predict the targeted binding relationship between circ_UBE2D2 and miR-370-3p. (**C**) StarBase predicted that NR4A3 have targeted binding sites with miR-370-3p. (**D-E**) The dual-luciferase reporter assay was used to verify the targeted binding relationship between circ_UBE2D2 and miR-370-3p and between miR-370-3p and NR4A3. (**F**) The effect of circ_UBE2D2 on the expression of miR-370-3p was detected by qRT-PCR. (***p < 0.001, vs. Control; ^+++^*p* < 0.001, vs. LPS+shNC; ^^^*p* < 0.001, vs. MC).

All measurement data were described by mean ± standard deviation. Data in Fig. 1A, Figs. 2D-2E were analyzed by independent sample *t* test, and one-way analysis of variance (ANOVA) was adopted for inter-group comparisons. All statistical analyses were implemented by Graphpad 8.0 software, and *p* < 0.05 was considered statistically significant.

## Results

### Effects of circ_UBE2D2 on LPS-Induced Renal Tubular Epithelial Cell Viability and Apoptosis

In order to explore the effect of circ_UBE2D2 on LPS-induced renal tubular epithelial cell damage, we first detected the expression of circ_UBE2D2 in LPS-induced HK-2 cell damage. It was found that the expression level of circ_UBE2D2 in LPS-induced HK-2 cells overtly exceeded that in untreated cells (Fig. 1A). Besides, the expression of circ_UBE2D2 in cells transfected with circ_UBE2D2 silencing vector was detected to be signally reduced in LPS-induced HK-2 cells (Fig. 1B), which confirmed that our transfection experiment was successful. Therefore, follow-up biological function research can be carried out. Initially, through MTT assay, the effect of circ_UBE2D2 on LPS-induced cell viability was determined. The data revealed that circ_UBE2D2 silencing could impair the effect of LPS-induced on declining HK-2 cell viability (Fig. 1C). According to the findings of flow cytometry, circ_UBE2D2 silencing was discovered to inhibit LPS-induced apoptosis (Fig. 1D). Moreover, western blot was utilized to assess the effect of circ_UBE2D2 on LPS-induced HK-2 cell apoptosis-related protein and NR4A3 expressions. It was noticed that circ_UBE2D2 silencing could inhibit NR4A3, Bax and Cleaved caspase-3 expressions that had been up-regulated by LPS, and promote Bcl-2 expression that had been down-regulated by LPS (Fig. 1E).

### Prediction Analysis of circ_UBE2D2 Target Gene

StarBase and miRDB were introduced to predict the binding miRNAs of circ_UBE2D2 and NR4A3, respectively, and the co-targeting miRNA with the highest score (miR-370-3p) was selected as the research object (Fig. 2A). Then, StarBase was used to predict the targeted binding relationship between circ_UBE2D2 and miR-370-3p, and the genes that have targeted binding sites with miR-370-3p. The results indicated that circ_UBE2D2 and miR-370-3p had a targeted binding relationship, and NR4A3 was the target gene of miR-370-3p (Figs. 2B and 2C). Furthermore, the dual-luciferase reporter assay was conducted to verify the targeted binding relationship between circ_UBE2D2 and miR-370-3p and between miR-370-3p and NR4A3. After co-transfection of miR-370-3p mimic with circ_UBE2D2-WT or NR4A3-WT into cells, the luciferase activity of cells was observed to be lessened; however, co-transfection of miR-370-3p mimic with circ_UBE2D2-MUT or NR4A3-MUT barely affected the luciferase activity of cells (Figs. 2D and 2E). Next, the effect of circ_UBE2D2 on the expression of miR-370-3p was detected by qRT-PCR, uncovering that circ_UBE2D2 silencing could attenuate the inhibitory effect of LPS on miR-370-3p expression (Fig. 2F).

### Effects of circ_UBE2D2 on LPS-Induced Renal Tubular Epithelial Cell Viability and Apoptosis by Targeting miR-370-3p/NR4A3

**Fig. 3:**
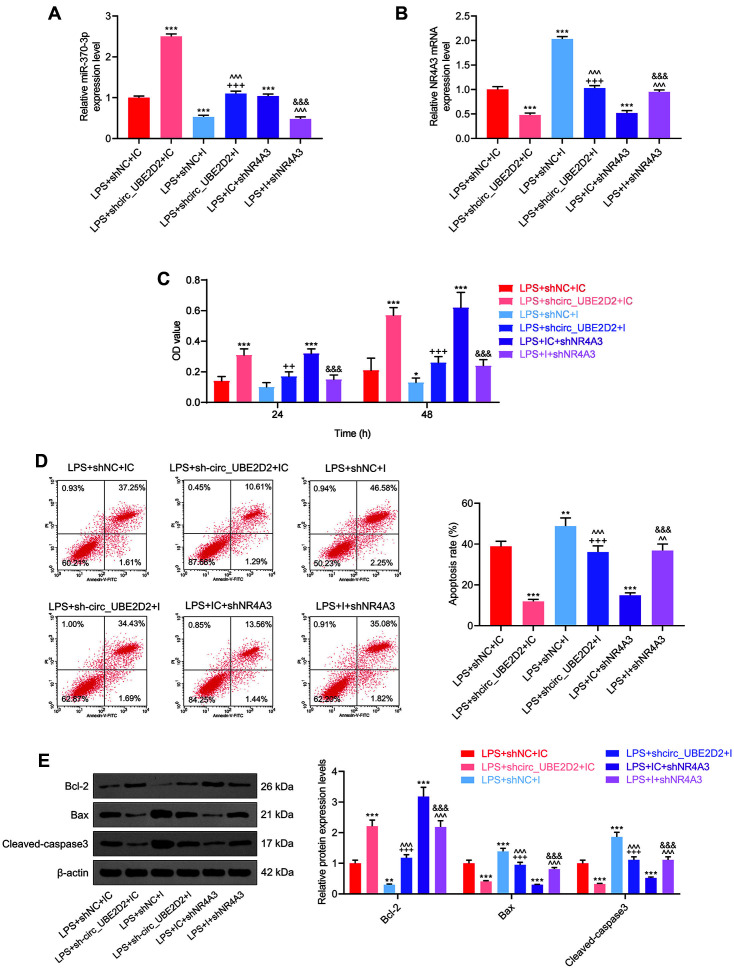
Effects of circ_UBE2D2 on LPS-induced renal tubular epithelial cell viability and apoptosis by targeting miR-370-3p/NR4A3. (**A**) The expression of miR-370-3p in the transfected cells was detected by qRT-PCR. (**B**) The expression of NR4A3 in the transfected cells was quantified by qRT-PCR. (**C**) MTT assay was conducted to detect cell viability. (**D**) Flow cytometry was used to measure apoptosis. (**E**) Western blot was exploited to quantitate the expressions of apoptosis-related proteins in each group. (*p < 0.05, **p < 0.01, ***p < 0.001, vs. LPS+shNC+IC; ^++^*p* < 0.01, ^+++^*p* < 0.001, vs. LPS+sh-circ_UBE2D2+IC; ^^*p* < 0.01, ^^^*p* < 0.001, vs. LPS+shNC+I; ^&&&^*p* < 0.001, vs. LPS+IC+shNR4A3).

To fathom out the effect of circ_UBE2D2 targeting miR-370/NR4A3 on the biological functions of LPS-induced HK-2 cells, sh-circ_UBE2D2, shNR4A3 and miR-370 inhibitor were transfected into the cells. Firstly, the expressions of NR4A3 and miR-370 in the transfected cells were detected by qRT-PCR, confirming the success of transfection experiment where miR-370-3p inhibitor diminished miR-370-3p expression, and shNR4A3 reduced NR4A3 expression (Figs. 3A and 3B). Furthermore, cell viability was evaluated through MTT assay, the results of which affirmed that miR-370-3p inhibitor reversed the promoting role of circ_UB2D2 silencing in cell viability, and shNR4A3 offset such effect of miR-370-3p inhibitor (Fig. 3C). Thereafter, cell apoptosis was gauged via flow cytometry. The findings revealed that miR-370-3p inhibitor neutralized the suppressing effect of circ_UB2D2 silencing on cell apoptosis, and shNR4A3 weakened the facilitating effect of miR-370-3p inhibitor on cell apoptosis (Fig. 3D). In line with the data of western blot, miR-370-3p inhibitor reversed the suppression of Bax and Cleaved caspase-3 expressions by circ_UB2D2 silencing and the promotion of Bcl-2 expression, while the reversing effect of miR-370-3p inhibitor was impaired by shNR4A3 (Fig. 3E).

### Effects of circ_UBE2D2 on Renal Tissue Injury on CLP Model Rats

**Fig. 4:**
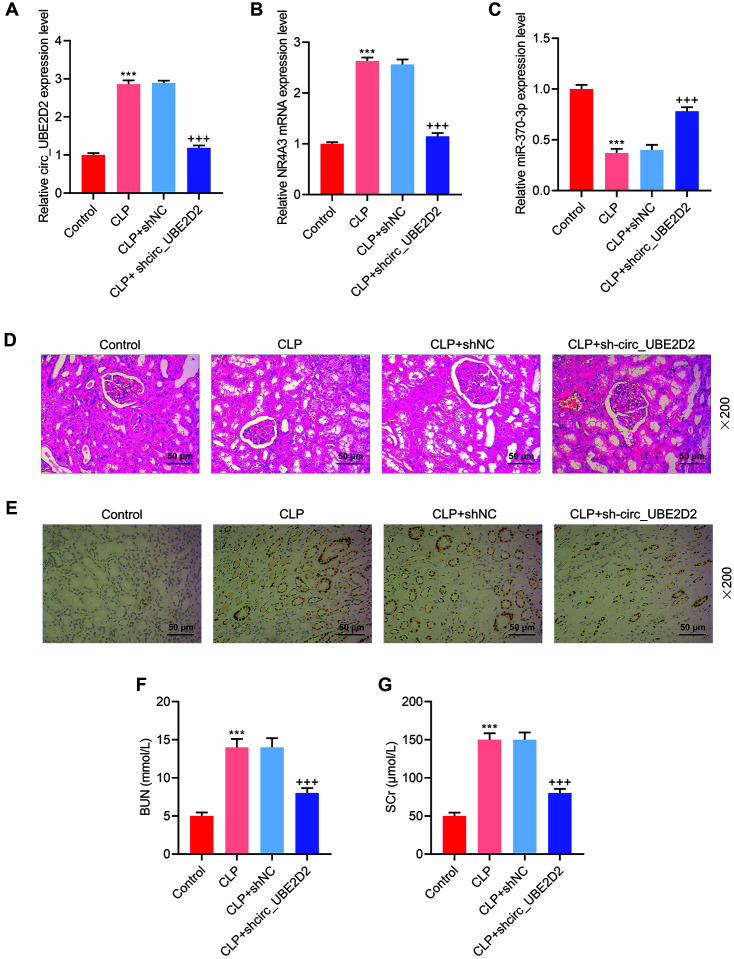
Effects of circ_UBE2D2 on renal tissue injury in CLP rat model. (**A-C**) Sh-circ_UBE2D2 lentivirus was injected into CLP rats, and then qRT-PCR was used to detect the expressions of circ_UBE2D2, NR4A3 and miR-370-3p in the rat kidney tissue of each group. (**D**) HE staining was conducted to observe the damage of the kidney tissue of CLP rats that were injected with sh-circ_UBE2D2 lentivirus. (**E**) TUNEL staining was conducted to observe the cell apoptosis in CLP rats that were injected with sh-circ_UBE2D2 lentivirus. (**F-G**) The levels of blood urea nitrogen (BUN) and serum creatinine (SCr) were measured in rat serum of each group. (***p < 0.001, vs. Control; ^+++^*p* < 0.001, vs. CLP+shNC).

To further confirm the results obtained through in vitro experiments, the CLP rat model was constructed to conduct in vivo experiments and verify the above results. First of all, sh-circ_UBE2D2 lentivirus was injected into CLP rats, and then qRT-PCR was implemented to quantitate the expressions of circ_UBE2D2, NR4A3 and miR-370-3p in the rat kidney tissue of each group. It could be noted that circ_UBE2D2 expression in CLP rats was elevated, and then diminished under the injection of sh-circ_UBE2D2 lentivirus (Fig. 4A). Moreover, sh-circ_UBE2D2 also inhibited the up-regulation of NR4A3 in the CLP rats (Fig. 4B). Moreover, sh-circ_UBE2D2 promoted miR-370-3p expression in the CLP rats (Fig. 4C). The damage of the rat kidney tissue of each group was observed through HE staining. In the control rats, the kidney tissue was normal. However, the kidney tissue of CLP rats presented vacuolar degeneration and bleeding, while that of CLP rats injected with sh-circ_UBE2D2 lentivirus exhibited less vacuolar degeneration (Fig. 4D). Through TUNEL staining, the positive cells in the CLP rats were observed to be markedly increased compared to those in control rats, while the positive cells in CLP rats injected with sh-circ_UBE2D2 lentivirus were notably reduced. In addition, the kidney injury marker (BUN and Scr) levels were measured, and the results showed that BUN and Scr levels were higher in the serum of CLP rats than in the serum of control rats, but circ_UBE2D2 silencing dwindled the levels of BUN and Scr in the serum of CLP rats (Figs. 4F and 4G).

## Discussion

CircRNAs are a new type of endogenous non-coding RNA that can competitively bind to miRNA as competitive endogenous RNA (ceRNA) to regulate gene expression [27-29]. Previous studies have revealed the role of some circRNAs in SAKI [30-32]. For instance, Garcia *et al*. described the role of circRNA in sepsis specifically and discussed the feasibility of circRNA as a biomarker for sepsis diagnosis [32]. More and more evidence also suggested that the misregulation of circRNA is an early event of sepsis [33]. Circ_UBE2D2 has targeted binding sites with miRNAs involved in SAKI process; however, the role of circ_UBE2D2 in SAKI has not been clarified, and whether circ_UBE2D2 can affect SAKI process through endogenous competitive binding with miRNAs deserves further investigation. In our study, we detected abnormal up-regulation of circ_UBE2D2 in LPS-induced HK-2 cells, which rendered us more skeptical about the possibility of circ_UBE2D2 playing a biological role in the progression of SAKI. Then, we constructed circ_UBE2D2 silencing vector and transfected it into SAKI cells, and unveiled that sh-circ_UBE2D2 could enhance SAKI cell viability and inhibit apoptosis, initially revealing the role of circ_UBE2D2 in SAKI.

In order to further expound the influence of circ_UBE2D2 on SAKI process by targeting downstream genes, bioinformatics analyses were applied and predicted that miR-370-3p and circ_UBE2D2 had a strong targeted binding relationship. Chen *et al*. identified four pairs of miRNA-mRNAs associated with sepsis, including miR-370-3p, through bioinformatics analysis [34]. In addition, it has also been confirmed that paclitaxel-dominated gene regulatory axis makes impacts upon promoting the chemotherapy effect of paclitaxel in alleviating SAKI [18]. Furthermore, long chain genes lncRNA NEAT1 and Lnc-MALAT1 can modulate the progression of sepsis through sponging miR-370-3p [35-37]. In this study, miR-370-3p inhibitor reversed the effects of circ_UB2D2 silencing on promoting cell viability and inhibiting cell apoptosis. Based on the above findings, circ_UBE2D2 was demonstrated to be capable of impacting SAKI progression via targeting miR-370-3p.

Furthermore, studies have proven that NR4A3 is a pro-apoptotic gene that is strongly expressed in AKI [22, 23]. Based on StarBase prediction results, we found that circ_UBE2D2 and NR4A3 both can bind to miR-370-3p. Hence, we speculated that circ_UBE2D2 may influence the process of renal injury in sepsis by targeting miR-370-3p to promote NR4A3 expression. In accordance with our conjecture, shNR4A3 offset the impacts of miR-370-3p inhibitor on inhibiting cell viability and promoting apoptosis. In addition, to verify the effects of circ_UBE2D2/miR-370-3p/NR4A3 regulatory axis on the cellular level, we established the SAKI rat model, and the results corroborated that circ_UBE2D2 could still affect the expressions of miR-370-3p and NR4A3 in vivo. Circ_UBE2D2 silencing ameliorated renal tissue injury and apoptosis in SAKI rats.

All in all, the study authenticates the positive role of circ_UBE2D2/miR-370-3p/NR4A3 regulatory axis in SAKI progression and demonstrates that silencing circ_UBE2D2 can promote cell viability while inhibiting cell apoptosis in SAKI by targeting miR-370-3p/NR4A3 axis, which provides a new possible gene target for SAKI treatment.

## Figures and Tables

**Table 1:** Primers used in qRT-PCR analysis.

Genes	Primer sequences
circ_UBE2D2	Forward: 5'- AATGGCAGCATTTGTCTTGA-3' reverse: 5'- GCCCCTGTGAGTAAGCTACG -3'
MiR-370-3p	Forward: 5'- GGTGTCGTATCCAGTGCAATTG -3' reverse: 5'- GTCGTATCCAGTGCGTGTCG -3'
NR4A3	Forward: 5'- TGCGTCCAAGCCCAATATAGC-3' reverse: 5'- GGTGTATTCCGAGCTGTATGTCT -3'
β-actin	Forward: 5'- GAGCGAGCTCAATGAGTTTGAGAGGTTTG -3' reverse: 5'- CTAGTCTAGACAATTTAGAACTAAACTCCAAC -3'
U6	Forward: 5'- GCTTCGGCAGCACATATACTAAAA -3' reverse: 5'- CGCTTCACGAATTTGCGTGTCAT -3'
